# Impact of Exclusive Adjuvant Radiotherapy on Fatigue and Quality of Life in Breast Cancer: A Prospective Clinical Study

**DOI:** 10.3390/cancers18162532

**Published:** 2026-08-07

**Authors:** Jesús Baltasar González-Rubino, Rocío Martín-Valero, Francisco José Vera-Serrano, Ismael García-Campanario, Francisco Javier Martin-Vega, Maria Jesus Vinolo-Gil

**Affiliations:** 1 Rehabilitation Clinical Management Unit, Hospital Punta Europa, Campo de, Oeste Health District, 11202 Algeciras, Spain; 2 Department of Physiotherapy, Faculty of Health Science, University of Malaga, 29071 Malaga, Spain; rovalemas@uma.es; 3 Rehabilitation Clinical Management Unit, Interlevels-Intercenters Hospital Puerta del Mar, Hospital Puerto Real, Cadiz Bay-La Janda Health District, 11006 Cadiz, Spain; francisco.vera@uca.es; 4 Department of Medicine and Surgery, Faculty of Medicine, University of Cadiz, 11003 Cadiz, Spain; ismael.garcia@uca.es; 5 Department of Nursing and Physiotherapy, University of Cadiz, 11003 Cadiz, Spain; javier.martin@uca.es; 6 Research Unit, Institute for Biomedical Research and Innovation Institute of Cadiz (INiBICA), Puerta del Mar University Hospital, University of Cadiz, 11003 Cadiz, Spain

**Keywords:** breast cancer, radiotherapy, cancer-related fatigue, quality of life, physical activity

## Abstract

**Background/Objectives**: Breast cancer treatments, including adjuvant radiotherapy, significantly improve survival but are associated with cancer-related fatigue (CRF), a debilitating symptom that severely impacts quality of life. Previous studies often evaluate fatigue by combining radiotherapy with systemic therapies, confounding its independent association. This study aims to isolate the specific relation between exclusive radiotherapy and the longitudinal progression of fatigue in surgically treated breast cancer patients. **Methods**: A longitudinal study was conducted in a cohort of 70 women with breast cancer who underwent breast-conserving surgery and subsequent adjuvant radiotherapy. Cancer-related fatigue levels were evaluated across behavioral, affective, sensory, and cognitive dimensions using the Piper Fatigue Scale-Revised (PFS-R), while global health status and quality of life (QoL) were measured using the EORTC QLQ-C30 questionnaire, both before and after radiotherapy. Data were analyzed using generalized linear mixed-effects models. **Results**: Following radiotherapy, a marked and statistically significant increase was observed in total fatigue (1.61 ± 1.16 vs. 5.37 ± 2.76; *p* < 0.001) and across all specific domains (*p* < 0.001), accompanied by a decline in global health status (82.6 ± 20.2 to 60.1 ± 29.2). Assessment time (pre- vs. post-RT) was identified as the factor most strongly associated with an increase in fatigue. However, better baseline global health status significantly attenuated the increase in total, behavioral, affective, and sensory fatigue (time × QoL interaction, *p* < 0.001). Furthermore, higher physical activity levels were significantly associated with lower behavioral fatigue (*p* = 0.042) and showed a protective trend for sensory (*p* = 0.055) and total fatigue (*p* = 0.092). **Conclusions**: Radiotherapy is strongly associated with a significant increase in cancer-related fatigue scores across all dimensions. Better baseline quality of life acts as a protective factor that is associated with attenuated fatigue progression, while regular physical activity correlates with a mitigated impact, particularly within the behavioral domain, although it did not reach statistical significance across all fatigue dimensions. These findings highlight the critical need for routine clinical monitoring of fatigue and strongly support the implementation of structured, exercise-based interventions during adjuvant treatment.

## Simple Summary

Breast cancer is the most common cancer in women, and while treatments like radiotherapy improve survival, they are often associated with severe fatigue that reduces overall quality of life. Previous studies usually evaluated fatigue by mixing various treatments, making it difficult to understand the specific role of radiotherapy alone. This study isolated this relationship by analyzing fatigue levels and overall well-being exclusively in breast cancer patients receiving adjuvant radiotherapy after surgery. The results showed a severe increase across all dimensions of fatigue and a sharp decline in quality of life after treatment. Crucially, having a better initial quality of life and engaging in regular physical activity acted as protective factors, which were associated with a reduced severity of these side effects. These findings highlight the urgent need for routine fatigue screening and personalized exercise programs to safeguard the well-being of breast cancer patients during radiotherapy.

## 1. Introduction

Breast cancer (BC) remains the most frequently diagnosed malignancy and a leading cause of cancer-related mortality among women worldwide [1,2]. Despite the rising incidence of BC, mortality rates have not increased proportionally, reflecting significant advancements in early detection and modern multimodal therapies [3,4]. Consequently, the population of BC survivors is growing, shifting the clinical focus from mere survival to managing the persistent, long-term side effects associated with cancer treatments. As patients live longer post-treatment, improving their Health-Related Quality of Life (HRQoL) by addressing debilitating symptoms has become a major public health priority [5].

Among the most distressing and prevalent symptoms experienced by BC survivors is Cancer-Related Fatigue (CRF). Unlike ordinary tiredness, CRF is a persistent, subjective sense of physical, emotional, and cognitive exhaustion that is disproportionate to recent exertion and is not relieved by adequate rest [6]. CRF significantly impairs daily functioning and is a powerful predictor of overall life dissatisfaction [7,8]. Furthermore, long-term studies have even suggested that experiencing severe CRF at the end of treatment may be associated with poorer overall survival outcomes in women with cancer [9,10]. The etiology of CRF is multifactorial, yet it is intrinsically linked to the cancer trajectory itself and the aggressive nature of the required treatments [11].

Adjuvant radiotherapy (RT) is a standard and highly effective modality following conservative surgery in early-stage BC, significantly improving locoregional control [12,13]. However, RT is a known contributor to the symptom burden. The physiological load of radiation, combined with potential underlying factors such as localized inflammation, sleep fragmentation, and psychological distress, can trigger or exacerbate fatigue [14]. Research indicates that fatigue typically peaks during the course of breast irradiation, and while it often improves weeks or months after completion, a meaningful subset of patients experiences persistent fatigue [15]. Moreover, certain factors, such as psycholo-gical distress or the presence of comorbidities, make specific subgroups of patients more vulnerable to experiencing severe fatigue during RT [16].

While the relationship between multimodal BC treatments and fatigue is recognized, many studies analyze fatigue without isolating specific treatment modalities (e.g., combining surgery, chemotherapy, and hormonal therapy) [9,10]. There is a critical need to understand the isolated impact of radiotherapy on CRF and HRQoL. Additionally, evidence suggests that physical activity may act as a potent, non-pharmacological intervention to mitigate CRF by increasing energy levels, managing pain, and improving overall well-being [17,18]. Therefore, the primary objective of this study was to analyze the relationship between fatigue and radiotherapy exclusively in surgically treated women receiving RT as their sole active treatment. Secondary objectives included comparing fatigue levels before and after RT and evaluating the impact of pre-treatment physical activity and exploring the protective role of baseline global health status (HRQoL) to improve tailored care.

## 2. Materials and Methods

### 2.1. Study Design and Participants

A prospective observational epidemiological study was conducted at the Lymphedema Unit of Algeciras (Servicio Andaluz de Salud, Cádiz, Spain). The study population consisted of female patients diagnosed with breast cancer. The inclusion criteria were: (a) age over 18 years; (b) having undergone total or conservative mastectomy; and (c) undergoing their first course of radiotherapy exclusively, with no prior history of chemotherapy and with no initiation of endocrine therapy prior to the final post-radiotherapy assessment, in order to isolate the specific effect of radiation. Exclusion criteria comprised severe psychological or neurological disorders that could interfere with proper data collection, as well as involvement in active legal litigation.

All patients underwent standard adjuvant radiotherapy protocols following breast-conserving surgery. Based on individual clinical criteria and dose constraints, the prescribed treatment primarily consisted of either a moderately hypofractionated scheme (e.g., 40.05 Gy in 15 daily fractions) or an ultra-hypofractionated schedule (e.g., 26 Gy in 5 fractions) using 3D-Conformal Radiotherapy (3D-CRT) or Volumetric Modulated Arc Therapy (VMAT). In addition to whole-breast irradiation, a sequential or simultaneous integrated boost (SIB) was delivered to the surgical tumor bed where clinically indicated. Furthermore, regional nodal irradiation was not routinely administered; it was strictly limited to the minority of patients presenting with positive axillary lymph nodes (5.7%), in accordance with standard oncological guidelines. Adjuvant radiotherapy was initiated within a strict timeframe of 4 weeks to 3 months following surgical intervention. Finally, to isolate the specific effect of radiation, it was confirmed that no patients had initiated endocrine therapy prior to the post-radiotherapy assessment.

### 2.2. Sample Size Calculation

The priori sample size calculation was focused on the primary outcome of fatigue severity to ensure sufficient statistical power for longitudinal comparisons. To detect a minimum difference of 0.87 units in the Piper Fatigue Scale-Revised (PFS-R) scores between the two assessment time points using a two-sided paired t-test, assuming a standard deviation of 2.45, both parameters estimated based on the previously published literature [19,20], an alpha risk of 0.05, and a beta risk of 0.20 (80% statistical power), an optimal sample size was determined. Anticipating a 10% dropout rate, the target sample was established at 70 subjects. The calculation was performed using the pwr package in R software (v.4.2.2). Ultimately, a total of 70 patients were consecutively enrolled and analyzed, successfully reaching the optimal sample size required to maintain robust statistical validity.

### 2.3. Instruments and Variables

To comprehensively assess the clinical and functional impact of fatigue and its relationship with oncological treatment, data were collected using three validated instruments. Fatigue severity was evaluated using the Piper Fatigue Scale-Revised (PFS-R) [21]. This instrument provides a Total Fatigue score as well as scores across four specific multidimensional domains: behavioral (BEH), affective (AFF), sensory (SEN), and cognitive (COG). The European Organization for Research and Treatment of Cancer Quality of Life Questionnaire (EORTC-QLQ-C30) [22] was administered to assess the patients’ perceived global health status and overall quality of life (QoL) as well as specific symptom scales and functional domains (including pain, emotional, cognitive, and social interference). Additionally, self-reported physical activity levels were measured using the International Physical Activity Questionnaire (IPAQ) [23]. For the regression models, physical activity was derived from the standardized scoring of the IPAQ, classifying patients into three established categories: low, moderate, and high. For baseline demographic reporting purposes only, this was collapsed into a descriptive binary variable (regular physical activity: Yes [moderate/high]/No [low]).

For the descriptive analyses and statistical modeling, variables were defined and categorized as follows. To provide a detailed baseline characterization of the study sample, specific sociodemographic and clinical variables were collected. Sociodemographic data included age, marital status, educational level, employment status, smoking habits, basic anthropometric measures (weight, height, and body mass index [BMI]), and general engagement in sporting activities. Clinical variables evaluated comprised the specific histological type of breast cancer (e.g., ductal, infiltrating, in situ, Paget), TNM staging, time since diagnosis, and the type of surgical treatment received (e.g., conservative surgery).

In the context of the regression models, the dependent variables (primary outcomes) were Total Fatigue and its four constituent domains (behavioral, affective, sensory, and cognitive), all modeled as continuous variables. The independent variables (primary predictors) included: (1) treatment time point, defined as a categorical variable evaluating the moment of assessment (pre-radiotherapy vs. post-radiotherapy); (2) physical activity level, derived from the IPAQ and defined as a categorical variable with three levels (low, moderate, and high); and (3) global health status (QoL), modeled as a continuous variable reflecting the patients’ overall perceived health status. While all specific EORTC-QLQ-C30 symptom subscales were analyzed descriptively to characterize the clinical evolution (pre- vs. post-radiotherapy), only the Global Health Status/QoL score was selected as an independent variable in the regression models. This approach was chosen to ensure model parsimony and avoid statistical over-fitting due to multiple testing.

### 2.4. Procedure

Eligible patients were recruited consecutively during their initial consultation with a Physical Medicine and Rehabilitation specialist. This was a strictly observational study; therefore, the role of the Physical Medicine and Rehabilitation department was focused entirely on clinical assessment and questionnaire administration, with no physical interventions applied during the study period. After obtaining written informed consent, a trained physiotherapist administered the questionnaires either face-to-face or via telephone. Clinical data were extracted from the patients’ electronic medical records. Longitudinal assessments were performed at two distinct time points: at the initiation of radiotherapy treatment (T1, conducted within the week prior to the first session) and upon completion of the radiotherapy sessions (T2, conducted within the week following the last session).

### 2.5. Statistical Analysis

Descriptive statistics were calculated to characterize the study sample. Continuous variables were expressed as means and standard deviations (SD), or medians and interquartile ranges (IQR) where appropriate. Categorical variables were presented as percentages along with their 95% confidence intervals (95% CI).

To determine the effect of treatment time point (pre- vs. post-radiotherapy), physical activity level, and global health status on fatigue, both globally and within its behavioral, affective, sensory, and cognitive domains, mixed-effects regression models were employed, following the methodology proposed by Zuur and Ieno [24]. Missing data resulting from patient dropout were handled implicitly within the mixed-effects framework using maximum likelihood estimation, which accounts for incomplete longitudinal profiles without requiring imputation.

Initially, a saturated model including all aforementioned factors and their second-degree interactions was fitted. Different error distributions (Gaussian with an identity link function; Gamma with a log link function) were evaluated. The best fit was determined by selecting the model with the lowest Akaike Information Criterion (AIC) and the best residual compliance with normality and homoscedasticity assumptions. Subsequently, the random structure of the model [1 | ID] was optimized using the lme4 (v.1.1-30) [25] and lmerTest (v.3.1-3) packages [26].

Finally, the fixed-effects structure was optimized through backward selection by comparing nested models using the AIC. Model interpretation was performed by inspecting the coefficients and calculating the ANOVA with the car package (v.3.1-3) [27]. The effects of fixed factors were further examined by calculating estimated marginal means and applying Tukey’s post hoc test using the emmeans package (v.1.10.7) [28].

All statistical analyses were conducted using R software (v.4.2.2) [29]. Graphical representations were generated using the ggplot2 (v.4.0.0) [30] and ggpubr (v.0.6.0) [31] packages. The statistical significance level for all hypothesis testing was set at *p* < 0.05.

### 2.6. Ethical Considerations

The study was conducted in accordance with the ethical principles of the Declaration of Helsinki and was approved by the Clinical Research Ethics Committee (CEIC) of the province of Cádiz (Registration Code: PEIBA 138.20/No. 138.28). Strict confidentiality of patient data was maintained in compliance with the European General Data Protection Regulation (GDPR 2016/679) and the Spanish Organic Law 3/2018 on the Protection of Personal Data. Written informed consent was obtained from all participants prior to data collection. Detailed descriptive statistics and the full statistical report, along with the E-thics Committee Certificate, are available in the Supplementary Materials (File S1).

## 3. Results

### 3.1. Participants Characteristics

**Figure 1:**
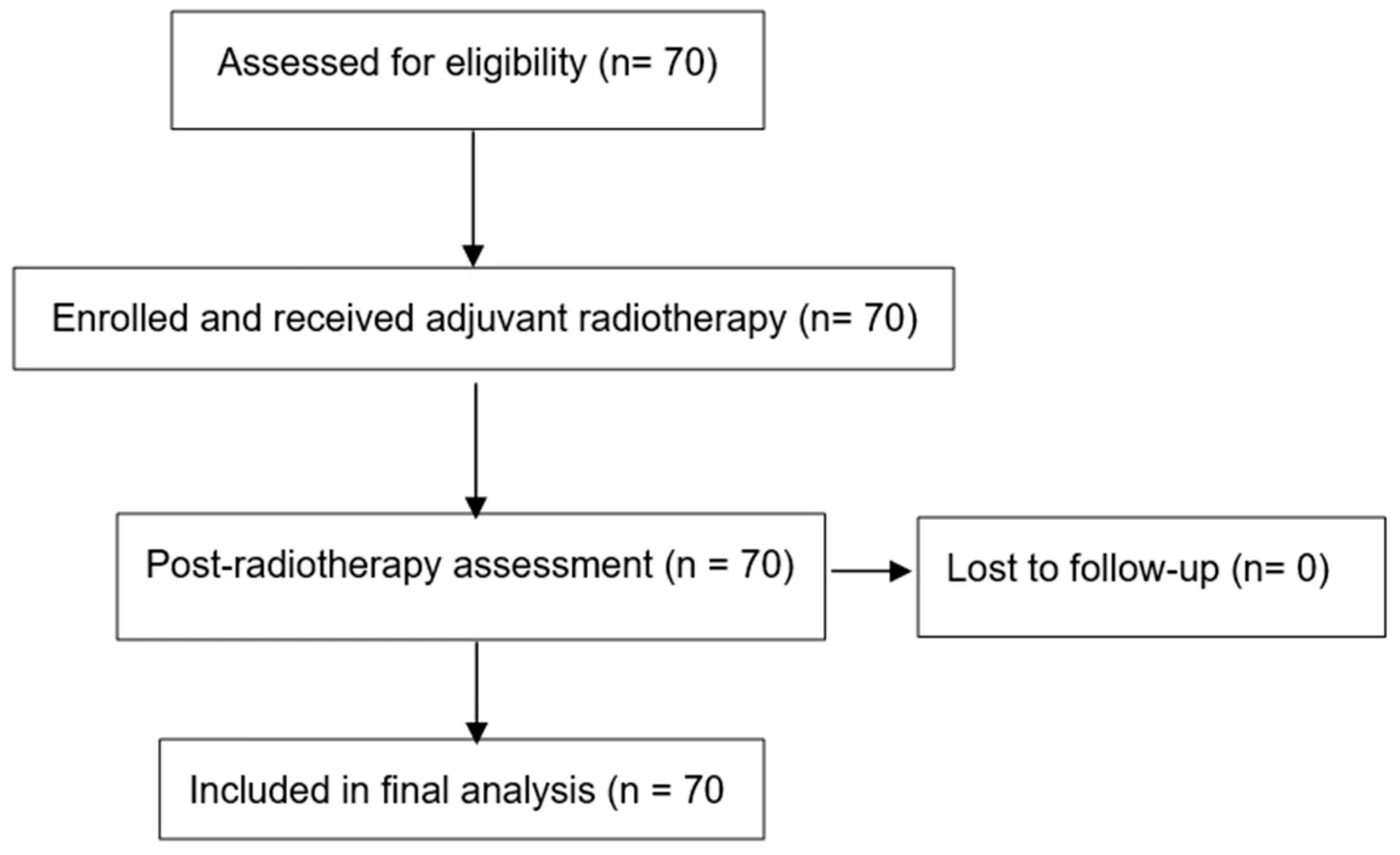
Patient flow diagram illustrating the screening, enrollment, and follow-up phases of the study participants.

A total of 70 women with surgically treated breast cancer receiving adjuvant radiotherapy were included (Figure 1). All enrolled patients successfully completed both the baseline (pre-radiotherapy) and post-radiotherapy assessments, resulting in no missing data or loss to follow-up for the primary outcome. Mean age was 55.8 ± 9.4 years. Most participants were married or living with a partner (77.1%), had completed secondary education (42.9%), and were employed (55.7%). Approximately 61.4% reported regular physical activity, whereas 75.7% were non-smokers.

Mean body mass index was 26.2 ± 7.3 before radiotherapy and 26.7 ± 5.7 after treatment. Invasive ductal carcinoma was the predominant histological subtype (98.6%), and all patients underwent breast-conserving surgery (Table 1).

### 3.2. Changes in Fatigue Following Radiotherapy

Comparison of pre- and post-radiotherapy assessments demonstrated a consistent and clinically significant increase in cancer-related fatigue across all dimensions assessed by the Piper Fatigue Scale-Revised. As detailed in Table 2, total fatigue and its specific dimensions (behavioral, affective, sensory, and cognitive) exhibited substantial worsening following treatment, all presenting large effect sizes (*p* < 0.001).

**Table 2:** Changes in fatigue and quality of life before and after radiotherapy.

Variable	Pre-Radiotherapy	Post-Radiotherapy	Mean Difference	Effect Size (Cohen’s d)	*p*-Value
Total fatigue (PFS-R)	1.61 ± 1.16	5.37 ± 2.76	3.76	1.78	<0.001 *
Behavioral fatigue	1.56 ± 1.10	5.42 ± 2.93	3.86	1.74	<0.001 *
Affective fatigue	1.67 ± 1.41	5.69 ± 2.90	4.02	1.76	<0.001 *
Sensory fatigue	1.60 ± 1.22	5.35 ± 2.82	3.75	1.73	<0.001 *
Cognitive fatigue	1.61 ± 1.18	5.08 ± 2.90	3.47	1.57	<0.001 *
Global health status (EORTC QLQ-C30)	82.62 ± 20.15	60.12 ± 29.18	−22.50	−0.90	<0.001 *
Global quality of life	5.96 ± 1.21	4.60 ± 1.76	−1.36	−0.90	<0.001 *

Values are expressed as mean ± SD. PFS-R: Piper Fatigue Scale-Revised. Effect sizes represent standard Cohen’s d (values ≥ 0.8 indicate a large effect).* Statistically significant at *p* < 0.001.

Likewise, radiotherapy was associated with increased fatigue severity and greater interference with occupational, social, sexual, and leisure activities, together with higher levels of weakness, need for rest, and functional limitations.

Parallel to the increase in fatigue, Health-Related Quality of Life (HRQoL), as assessed by the EORTC QLQ-C30, experienced significant deterioration following radiotherapy. The global health status/QoL score exhibited a marked decline from baseline to post-treatment (*p* < 0.001). This deterioration was also reflected in the specific functional domains of the questionnaire, where patients reported a noticeable reduction in physical, role, and social functioning after completing their radiation sessions. Symptom scales correspondingly worsened, primarily driven by radiation-induced side effects. However, as demonstrated by our interaction models, patients presenting with a higher baseline global QoL showed a significant protective effect, effectively buffering the progression and severity of fatigue across multiple dimensions.

### 3.3. Association Between Radiotherapy and Fatigue Domains

Generalized linear mixed-effects models identified assessment time (pre- versus post-radiotherapy) as the factor most strongly associated with the strongest with fatigue across all evaluated domains (*p* < 0.001). The original ANOVA summary supporting the omnibus p-values is available in Supplementary Materials (Table S1). To illustrate the clinical magnitude of these associations, the model effect estimates and their 95% Confidence Intervals (CIs) for all predictors are comprehensively detailed in Table 3.

**Table 3:** Mixed-effects models evaluating factors associated with cancer-related fatigue (Estimates and 95% CIs).

Factor/Predictor	Behavioral	Affective	Sensory	Cognitive	Total Fatigue
**Time (Pre/Post RT)**	Est. 1.79 [1.25–2.32] *p* < 0.001	Est. 1.58 [1.03–2.13] *p* < 0.001	Est. 1.54 [0.99–2.10] *p* < 0.001	Est. 0.83 [0.66–1.01] *p* < 0.001	Est. 1.49 [0.96–2.02] *p* < 0.001
**Quality of Life**	Est. −0.003 [−0.009–0.003] NS	Est. −0.006 [−0.012–−0.000] *p* < 0.05	Est. −0.005 [−0.011–0.001] NS	Est. −0.01 [−0.02–−0.008] *p* < 0.001	Est. −0.006 [−0.012–−0.001] *p* < 0.05
**Physical Activity Level**					
Low vs. Moderate	Est. −0.34 [−0.73–0.06] NS	Est. −0.10 [−0.51–0.31] NS	Est. −0.38 [−0.78–0.03] NS	Est. −0.09 [−0.50–0.31] NS	Est. −0.20 [−0.59–0.18] NS
Low vs. High	Est. −0.53 [−0.95–−0.10] *p* < 0.05	Est. −0.48 [−0.92–−0.03] *p* < 0.05	Est. −0.46 [−0.89–−0.03] *p* < 0.05	Est. −0.43 [−0.86–−0.002] *p* < 0.05	Est. −0.49 [−0.90–−0.08] *p* < 0.05
**Time × QoL**	Est. −0.01 [−0.02–−0.004] *p* < 0.01	Est. −0.01 [−0.02–−0.002] *p* < 0.05	Est. −0.01 [−0.02–−0.001] *p* < 0.05	--	Est. −0.008 [−0.014–−0.001] *p* < 0.05

Abbreviations: RT, radiotherapy; NS, not statistically significant. Values presented as Model Estimates (Est.) with 95% Confidence Intervals [CI].

For behavioral fatigue, significant main effects were observed for physical activity level and assessment time, alongside a significant interaction between time and global QoL. Post hoc analyses revealed that higher physical activity levels exerted a protective effect, being associated with lower behavioral fatigue. Additionally, better baseline global QoL significantly attenuated the sharp increase in behavioral fatigue following radiotherapy.

Regarding affective and sensory fatigue, significant effects were similarly found for assessment time, global QoL, and the interaction between both variables. Patients reporting better global health experienced a significantly smaller increase in these fatigue domains following radiotherapy. Furthermore, physical activity showed a borderline association (*p* = 0.055) with sensory fatigue, suggesting a trend toward lower sensory fatigue among more physically active women.

For cognitive fatigue, significant main effects were identified for assessment time and QoL, but no significant interaction was observed. Cognitive fatigue significantly worsened after radiotherapy, whereas better global health status was independently associated with lower cognitive impairment.

### 3.4. Overall Fatigue

**Figure 2:**
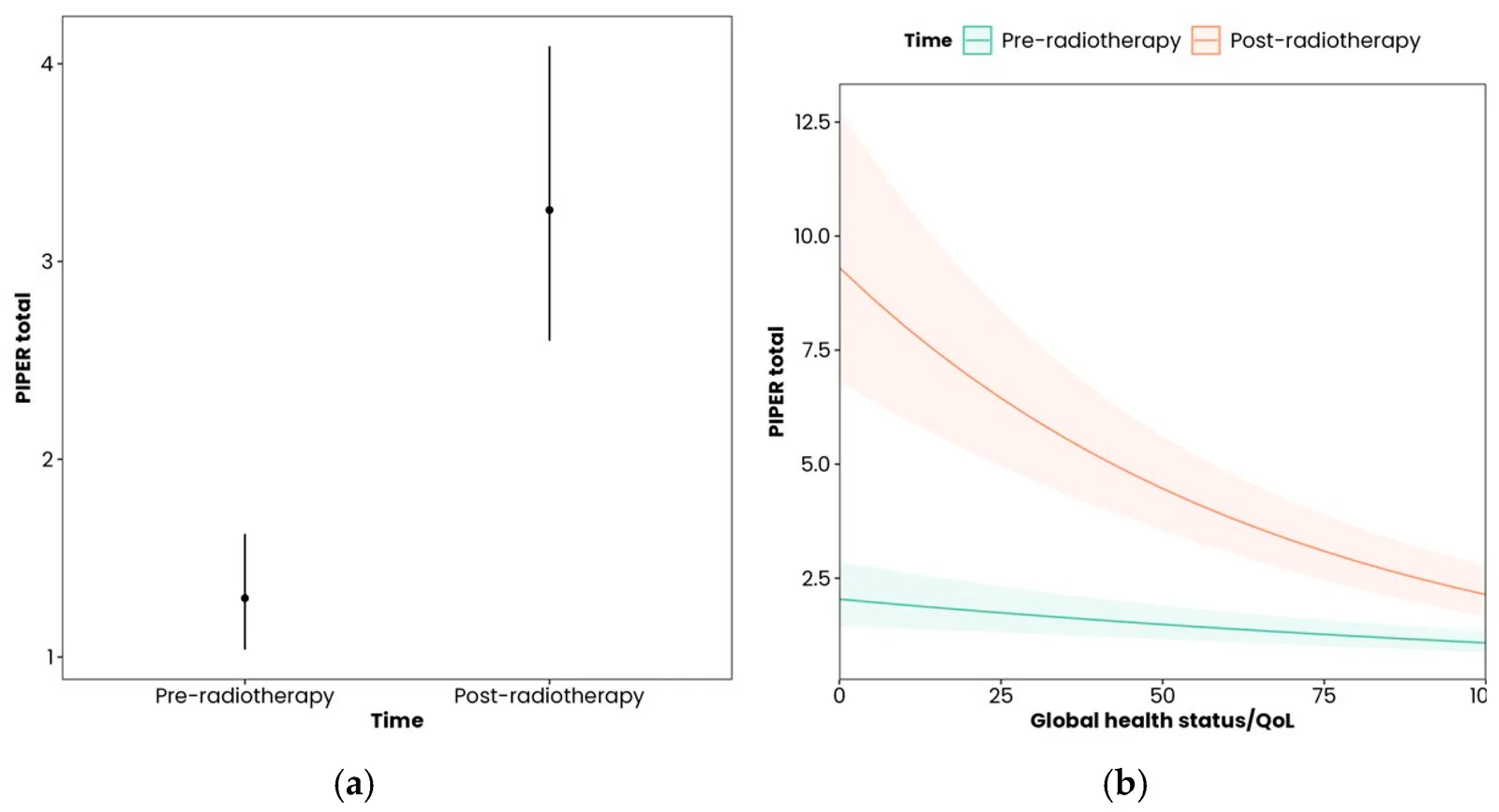
Longitudinal progression and protective factors of cancer-related fatigue. (**a**) Main effect of treatment time point on Total Fatigue (Piper Fatigue Scale-Revised), demonstrating a significant increase after exclusive adjuvant radiotherapy. (**b**) Interaction effect between Global Health Status/QoL (EORTC QLQ-C30) and treatment time on Total Fatigue, illustrating how a higher baseline quality of life significantly attenuates the post-radiotherapy fatigue spike. Vertical bars and shaded areas represent 95% confidence intervals. This figure illustrates Total Fatigue as a representative summary of the modeled effects; detailed statistical parameters for all individual fatigue domains are provided in Table 2 and Table 3.

The mixed-effects model for total fatigue revealed significant effects of assessment time, quality of life, and the interaction between time and quality of life (Table 3, Figure 2). Although physical activity did not reach statistical significance (*p* = 0.092), a consistent trend toward lower overall fatigue was observed among women reporting moderate or high physical activity levels.

Overall, these findings indicate that radiotherapy is associated with a marked increase in cancer-related fatigue across all evaluated dimensions. Better baseline quality of life, and to a lesser extent higher physical activity levels, appear to mitigate the magnitude of this increase (Table 4).

## 4. Discussion

The primary objective of this prospective clinical research was to analyze the relationship between cancer-related fatigue and radiotherapy exclusively in surgically treated breast cancer patients, thereby minimizing the independent association of radiation from the confounding impacts of prior chemotherapy or concurrent hormone therapy. In line with our secondary objectives, we evaluated the longitudinal progression of fatigue across two clinical milestones, examined the protective role of regular physical activity, and explored specific sociodemographic and clinical predictors to facilitate more tailored supportive care.

Unlike previous studies evaluating cancer-related fatigue in heterogeneous breast cancer populations [32,33,34], our cohort consisted exclusively of women who underwent breast-conserving surgery and received adjuvant radiotherapy without prior chemotherapy or concurrent endocrine therapy. This design minimized treatment-related confounding and enabled a more accurate characterization of the independent contribution of radiotherapy to fatigue development.

Importantly, by strictly isolating the radiotherapy effect prior to the onset of endocrine therapy, this study establishes a clean clinical baseline of acute toxicity in a real-world setting. This foundational evidence provides the necessary rationale for healthcare institutions to implement and justify early, targeted prehabilitation and rehabilitation programs [35].

Regarding the longitudinal trajectory of symptoms, our data demonstrated a significant, progressive increase in CRF from the initiation of radiotherapy (T1) to its completion (T2). From a clinical perspective, the transition from a baseline score of approximately 1.6 to 5.4 on the Total Fatigue scale of the PFS-R represents a substantial shift from mild, manageable fatigue to moderate-to-severe exhaustion [21]. Furthermore, the 22.5-point drop in global health status on the EORTC QLQ-C30 well exceeds the universally accepted minimally clinically important difference (MCID) threshold of 10 points [36], highlighting the severe practical impact of radiation-induced toxicity on patients’ daily lives. This cumulative advancement of fatigue fits the established dose-effect framework of radiation oncology. Unlike the cyclic and fluctuating peaks characteristically observed during chemotherapy, radiation observed during fatigue typically intensifies continuously as the total Gray (Gy) dose builds up over successive weeks [37]. This phenomenon is biologically driven by localized normal tissue damage and the subsequent systemic upregulation of pro-inflammatory cytokines (such as IL-1β, IL-6, and TNF-α). These peripheral inflammatory signals are capable of crossing the blood–brain barrier, altering central neural processing and autonomic regulation, which ultimately manifests as severe somatic fatigue [38].

These findings reinforce the concept that fatigue during exclusive radiotherapy is not merely a subjective symptom but a progressive biological response to cumulative radiation exposure [39]. The absence of chemotherapy-related toxicity in our cohort strengthens the interpretation that radiotherapy itself is substantially associated with fatigue progression.

In exploring the determinants of fatigue, our generalized linear mixed-effects models revealed that baseline Global Quality of Life acted as a robust protective factor. Patients reporting better global health status experienced a significantly smaller increase in multidimensional fatigue post-treatment. This suggests that pre-treatment clinical and functional well-being provides a physiological and emotional buffer, enhancing a patient’s resilience against the cumulative stress of radiation.

Furthermore, regarding lifestyle habits, our results revealed that regular physical activity acted as a vital protective factor. Higher activity levels were significantly associated with lower fatigue, specifically in the behavioral dimension (*p* = 0.042), while demonstrating a non-significant protective trend for sensory and overall fatigue. This outcome supports the clinical consensus surrounding the “exercise paradox” in oncology, where controlled energy expenditure is associated with mitigated systemic fatigue [35]. As demonstrated by large-scale meta-analyses [35,40], structured physical exercise modulates systemic inflammation and preserves skeletal muscle efficiency. Importantly, because physical activity represents modifiable behavior, this evidence provides additional support for integrating supervised exercise programs into routine radiotherapy care rather than considering exercise merely as a general lifestyle recommendation.”

While our findings show a pronounced increase in fatigue, it is important to note that some previous research presents contrasting results. For instance, a previous study has reported more stable fatigue trajectories during exclusive radiotherapy, suggesting that severe exhaustion is predominantly driven by prior systemic treatments (such as chemotherapy) rather than radiation alone [41]. Similarly, although we observed a protective trend regarding physical activity, a previous review has found that exercise interventions specifically during radiotherapy did not significantly improve fatigue compared to standard care, highlighting the need for highly personalized and supervised exercise prescriptions rather than general physical activity recommendations [42].

### 4.1. Clinical Implications

The findings of this study have several practical implications for the multidisciplinary management of women undergoing exclusive adjuvant radiotherapy after breast-conserving surgery. The identification of lower baseline quality of life and physical inactivity as predictors of more severe fatigue highlights the importance of early risk stratification before treatment initiation. Incorporating baseline functional assessments together with routine evaluation of lifestyle habits may help identify patients at greater risk of developing clinically significant fatigue, allowing timely implementation of supportive interventions throughout radiotherapy.

Finally, based on the acute functional decline observed in our cohort, we hypothesize that integrating structured exercise programs into routine radiotherapy care could be highly beneficial. Rather than being considered merely an optional lifestyle recommendation, we suggest that future clinical strategies should explore supervised physical activity as a targeted supportive intervention to help manage fatigue and preserve functional status during treatment.

### 4.2. Strengths and Limitations

A major methodological strength of this study is its rigorous prospective design and strict selection criteria. By including exclusively women who underwent breast-conserving surgery receiving radiotherapy as the sole active treatment, we minimized the confounding effects of prior chemotherapy and concurrent endocrine therapy. While large systematic reviews, such as the one by Ruiz-Casado et al. [43], note that the independent impact of specific oncological therapies on long-term fatigue remains inconsistent across the literature due to heterogeneous populations, our homogeneous cohort successfully isolated this effect. Consequently, the present data provide precise, clinically relevant evidence on the independent contribution of radiotherapy to cancer-related fatigue, supporting the implementation of targeted supportive care strategies in this specific population.

However, several limitations should be acknowledged. First, the single-center design and modest sample size (*n* = 70) may limit the generalizability of our findings. Additionally, our strict inclusion criteria (excluding patients with prior chemotherapy or concurrent endocrine therapy) successfully isolated the effects of radiation, but this highly selected population limits the generalizability of our findings to the broader, more heterogeneous breast cancer population, who typically undergo multimodal treatments and may present with more diverse histological subtypes.

Second, our study lacks a longer-term follow-up beyond the completion of the radiotherapy sessions. This short duration of follow-up is a notable limitation that prevents assessing long-term fatigue recovery, as evidence suggests that fatigue observed during radiotherapy may naturally improve or resolve in the weeks and months following treatment completion. In addition, fatigue was assessed at only two discrete time points (before and immediately after radiotherapy). Because fatigue often evolves and may peak dynamically during the weeks of treatment, the lack of interim assessments prevents us from mapping the exact trajectory of symptom exacerbation. Consequently, this short timeframe restricts our understanding of the chronicity of fatigue and the potential compounding effects of subsequent treatments, such as endocrine or other systemic therapies. Future longitudinal studies incorporating follow-ups at 6 and 12 months are necessary to properly evaluate these long-term dynamics.

Third, the reliance on self-reported questionnaires to assess quality of life and physical activity may introduce response and recall biases. Moreover, while we controlled for baseline quality of life and physical activity, residual confounding from unmeasured variables remains possible. We did not systematically record other potential confounding variables such as exact comorbidities, sleep disturbances, psychological distress, or analgesic use. Future studies should incorporate these variables to better stratify the multifactorial nature of cancer-related fatigue.

Furthermore, the observational nature of the study and the absence of a non-irradiated comparison cohort preclude drawing definitive causal inferences regarding the relationships between the identified predictors and fatigue progression. Lastly, the absence of objective inflammatory biomarkers prevents direct confirmation of the biological mechanisms underlying fatigue observed during radiotherapy, although these pathways are well-supported by the previous literature [38,44].

To address these limitations, future multicenter studies with larger cohorts are warranted. Incorporating objective measures of physical activity (e.g., actigraphy) and analyzing inflammatory biomarkers will be crucial to validate these findings, better stratify the multifactorial nature of cancer-related fatigue and further elucidate the precise psychobiological mechanisms driving treatment-related exhaustion.

## 5. Conclusions

In conclusion, this prospective study demonstrates that adjuvant radiotherapy in women with breast cancer undergoing breast-conserving surgery is strongly associated with a severe, clinically significant, and multidimensional increase in cancer-related fatigue, affecting the behavioral, affective, sensory, and cognitive domains. This deterioration coexists with a marked decline in patients’ global health status and overall quality of life.

Nevertheless, the findings identify key clinical modifiers with important practical implications. A higher baseline quality of life emerged as a robust protective factor, attenuating both the severity and progression of post-treatment fatigue. Likewise, regular physical activity was significantly associated with lower fatigue only in the behavioral dimension, while showing a non-significant protective trend for overall and sensory fatigue.

These results highlight the need to implement routine screening and assessment of cancer-related fatigue before the initiation of radiotherapy. Furthermore, based on the functional decline observed in our cohort, we hypothesize that the future integration of supervised, individualized exercise-based interventions, alongside early multidisciplinary supportive care, could serve as a valuable clinical strategy to mitigate the adverse effects of radiotherapy and preserve the overall well-being of breast cancer survivors.

## Figures and Tables

**Table 1:** Baseline characteristics of the study population.

Variable	Overall (*n* = 70)
**Age (years)**	55.8 ± 9.4
**Body mass index (kg/m^2^)**	26.2 ± 7.3
**Marital status**	
Married/Living with partner	77.1%
Single	11.4%
Divorced/Separated	5.7%
Widowed	5.7%
**Educational level**	
No formal education	5.7%
Primary education	30.0%
Secondary education	42.9%
University education	21.4%
**Employment status**	
Employed	55.7%
Unemployed	11.4%
Homemaker	32.9%
**Regular physical activity**	
Yes	61.4%
No	38.6%
**Smoking status**	
Current smoker	24.3%
Non-smoker	75.7%
**Histological subtype**	
Invasive ductal carcinoma	98.6%
Other histological types	1.4%
**Surgical treatment**	
Breast-conserving surgery	100%
**Tumor Stage**	
Stage II	98.6%
Stage III	1.4%
**Laterality**	
Left breast cancer	58.6%
Right breast cancer	41.4%
**Axillary Lymph Node Status**	
Negative	94.3%
Positive	5.7%

Data are presented as mean ± standard deviation (SD) or percentage (%).

**Table 4:** Summary of Key Findings and Clinical Implications.

Factor/Domain	Significant Effects (Mixed Models)	Main Finding	Clinical Implication
Radiotherapy (Time)	✔ All PFS-R domains	Strongly associated with increased fatigue across all PFS-R domains.	Major factor associated with cancer-related fatigue; highlights the need for routine monitoring.
Global Quality of Life	✔ Affective, Sensory, Cognitive, Total fatigue	Better baseline QoL attenuated fatigue progression after radiotherapy.	Acts as a crucial protective factor against treatment-induced fatigue.
Time × QoL Interaction	✔ Behavioral, Affective, Sensory, Total fatigue	The protective effect of QoL buffers the specific fatigue spike caused by radiotherapy.	Maximizing pre-treatment well-being is vital to mitigate treatment toxicity.
Physical Activity	✔ Behavioral fatigue *(Trend: Sensory and Total)*	Higher activity levels were associated with lower fatigue, particularly in the behavioral domain.	Strongly supports the implementation of early exercise-based interventions.
Cognitive Fatigue	✔ Time, ✔ QoL *(No interaction)*	Significantly worsened after radiotherapy without the interaction buffering effect.	Closely related to patients’ perceived health status; requires targeted assessment.

✔ Significant (*p* < 0.05).

## Data Availability

The data presented in this study are available on request from the corresponding author. The data are not publicly available due to privacy and ethical restrictions.

## Supplementary Materials

The following supporting information can be downloaded at: https://www.mdpi.com/article/10.3390/cancers18162532/s1, File S1: Descriptive Statistics Tables, Statistical Report, and Ethics Committee Certificate; Table S1: Analysis of Variance (ANOVA) summary for generalized linear mixed-effects models evaluating factors associated with cancer-related fatigue. References [24,25,26,27,28,29,31,45,46,47] are cited in the Supplementary Materials.

## Abbreviations

The following abbreviations are used in this manuscript:

BMI	Body Mass Index
CEI	Comité de Ética de la Investigación (Institutional Review Board/Ethics Committee)
CRF	Cancer-Related Fatigue
EORTC	European Organization for Research and Treatment of Cancer Quality of Life Questionnaire
FACIT-F	Functional Assessment of Chronic Illness Therapy-Fatigue
HRQoL	Health-Related Quality of Life
IPAQ	International Physical Activity Questionnaire
TNM	Tumor, Node, Metastasis classification system

## References

[1] Ahmad A. (2019). Breast Cancer Statistics: Recent Trends. Adv. Exp. Med. Biol..

[2] Siegel R.L., Miller K.D., Jemal A. (2015). Cancer Statistics, 2015. CA Cancer J. Clin..

[3] Colina Ruizdelgado Delgado F., Pilas Pérez M., Lora Pablos D. (2012). Análisis de la supervivencia del cáncer de mama durante el decenio 1999–2008 en un Hospital Público de Madrid. Rev. Esp. Salud Pública.

[4] Hortobagyi G.N., de la Garza Salazar J., Pritchard K., Amadori D., Haidinger R., Hudis C.A., Khaled H., Liu M.C., Martin M., Namer M. (2005). The Global Breast Cancer Burden: Variations in Epidemiology and Survival. Clin. Breast Cancer.

[5] Berger A.M., Mooney K., Alvarez-Perez A., Breitbart W.S., Carpenter K.M., Cella D., Cleeland C., Dotan E., Eisenberger M.A., Escalante C.P. (2015). Cancer-Related Fatigue, Version 2.2015. J. Natl. Compr. Cancer Netw..

[6] Rocha S.R., Santos M.C.L., de Lopes M.V.O., Rodrigues A.B., de Sousa V.E.C., de Aquino C.B.Q., Mendes C.R.S. (2018). Accuracy of the Defining Characteristics of the Nursing Diagnosis for Fatigue in Women under Radiotherapy. Rev. Bras. Enferm..

[7] Prue G., Rankin J., Allen J., Gracey J., Cramp F. (2006). Cancer-Related Fatigue: A Critical Appraisal. Eur. J. Cancer.

[8] Abrahams H.J.G., Gielissen M.F.M., Schmits I.C., Verhagen C.A.H.H.V.M., Rovers M.M., Knoop H. (2016). Risk Factors, Prevalence, and Course of Severe Fatigue after Breast Cancer Treatment: A Meta-Analysis Involving 12 327 Breast Cancer Survivors. Ann. Oncol..

[9] Heumann P., Benner A., Behrens S., Chang-Claude J., Seibold P. (2025). Prognostic Value of Cancer-Related Fatigue at the End of Radiotherapy for Overall Survival ≥ 10 Years in Women with Breast Cancer. Breast Cancer Res..

[10] Payne C., Larkin P.J., McIlfatrick S., Dunwoody L., Gracey J.H. (2013). Exercise and Nutrition Interventions in Advanced Lung Cancer: A Systematic Review. Curr. Oncol..

[11] Bower J.E., Wiley J., Petersen L., Irwin M.R., Cole S.W., Ganz P.A. (2018). Fatigue after Breast Cancer Treatment: Biobehavioral Predictors of Fatigue Trajectories. Health Psychol..

[12] Guerrero E., Ballesteros H., Torres L.F. (2018). Respuesta Clínica En Pacientes Con Tumores de Mama Tratadas Con Radioterapia Conformacional Postmastectomía En El Instituto Nacional de Cancerología, Colombia. Rev. Colomb. Cancerol..

[13] Huang X., Chen Y., Chen W., Zhang X., Wu C., Zhang C., Sun S., Wu J. (2017). Effect of Radiotherapy after Breast-Conserving Surgery in Older Patients with Early Breast Cancer and Breast Ductal Carcinoma in Situ: A Meta-Analysis. Oncotarget.

[14] Suesada M.M., de Andrade Carvalho H., de Albuquerque A.L.P., Salge J.M., Stuart S.R., Takagaki T.Y. (2018). Impact of Thoracic Radiotherapy on Respiratory Function and Exercise Capacity in Patients with Breast Cancer. J. Bras. Pneumol..

[15] dos Santos D.E., Rett M.T., Mendonça A.C.R., Bezerra T.S., de Santana J.M., da Silva Júnior W.M. (2013). Efeito Da Radioterapia Na Função Pulmonar e Na Fadiga de Mulheres Em Tratamento Para o Câncer de Mama. Fisioter. Pesqui..

[16] Tödt K., Engström M., Ekström M., Efverman A. (2022). Fatigue During Cancer-Related Radiotherapy and Associations with Activities, Work Ability and Quality of Life: Paying Attention to Subgroups More Likely to Experience Fatigue. Integr. Cancer Ther..

[17] Potthoff K., Schmidt M.E., Wiskemann J., Hof H., Klassen O., Habermann N., Beckhove P., Debus J., Ulrich C.M., Steindorf K. (2013). Randomized Controlled Trial to Evaluate the Effects of Progressive Resistance Training Compared to Progressive Muscle Relaxation in Breast Cancer Patients Undergoing Adjuvant Radiotherapy: The BEST Study. BMC Cancer.

[18] Meneses-Echávez J.F., González-Jiménez E., Ramírez-Vélez R. (2015). Effects of Supervised Exercise on Cancer-Related Fatigue in Breast Cancer Survivors: A Systematic Review and Meta-Analysis. BMC Cancer.

[19] Drouin J. (2002). Aerobic Exercise Training Effects on Physical Function, Fatigue and Mood, Immune Status, and Oxidative Stress in Subjects Undergoing Radiation Treatment for Breast Cancer. Ph.D. Thesis.

[20] Mock V. (2004). Evidence-Based Treatment for Cancer-Related Fatigue. J. Natl. Cancer Inst. Monogr..

[21] Reeve B.B., Stover A.M., Alfano C.M., Smith A.W., Ballard-Barbash R., Bernstein L., McTiernan A., Baumgartner K.B., Piper B.F. (2012). The Piper Fatigue Scale-12 (PFS-12): Psychometric Findings and Item Reduction in a Cohort of Breast Cancer Survivors. Breast Cancer Res. Treat..

[22] Aaronson N.K., Ahmedzai S., Bergman B., Bullinger M., Cull A., Duez N.J., Filiberti A., Flechtner H., Fleishman S.B., Haes J.C.J.M.D. (1993). The European Organization for Research and Treatment of Cancer QLQ-C30: A Quality-of-Life Instrument for Use in International Clinical Trials in Oncology. J. Natl. Cancer Inst..

[23] Craig C.L., Marshall A.L., Sjöström M., Bauman A.E., Booth M.L., Ainsworth B.E., Pratt M., Ekelund U., Yngve A., Sallis J.F. (2003). International Physical Activity Questionnaire: 12-Country Reliability and Validity. Med. Sci. Sports Exerc..

[24] Zuur A.F., Ieno E.N. (2016). A Protocol for Conducting and Presenting Results of Regression-Type Analyses. Methods Ecol. Evol..

[25] Bates D., Mächler M., Bolker B.M., Walker S.C. (2015). Fitting Linear Mixed-Effects Models Using Lme4. J. Stat. Softw..

[26] Kuznetsova A., Brockhoff P.B., Christensen R.H.B. (2017). LmerTest Package: Tests in Linear Mixed Effects Models. J. Stat. Softw..

[27] Fox J., Weisberg S. (2018). An R Companion to Applied Regression.

[28] Lenth R.V. (2021). Emmeans: Estimated Marginal Means, Aka Least-Squares Means. https://www.scirp.org/reference/referencespapers?referenceid=3437485.

[29] Schauberger P., Walker A. (2025). *Openxlsx: Read, Write and Edit Xlsx Files*; R Package Openxlsx Version 4.2.8.1. CRAN: Contributed Packages. https://cran.r-project.org/web/packages/openxlsx/index.html.

[30] Wickham H. (2016). ggplot2.

[31] Kassambara A. (2026). *Ggpubr: ‘ggplot2’ Based Publication Ready Plots*; R Package Ggpubr Version 0.6.3. CRAN: Contributed Packages. https://cran.r-project.org/web/packages/ggpubr/index.html.

[32] Maass S.W.M.C., Brandenbarg D., Boerman L.M., Verhaak P.F.M., de Bock G.H., Berendsen A.J. (2021). Fatigue among Long-Term Breast Cancer Survivors: A Controlled Cross-Sectional Study. Cancers.

[33] Joly F., Lange M., Dos Santos M., Vaz-Luis I., Di Meglio A. (2019). Long-Term Fatigue and Cognitive Disorders in Breast Cancer Survivors. Cancers.

[34] Ho R.T.H., Fong T.C.T., Cheung I.K.M. (2014). Cancer-Related Fatigue in Breast Cancer Patients: Factor Mixture Models with Continuous Non-Normal Distributions. Qual. Life Res..

[35] Osoba D., Rodrigues G., Myles J., Zee B., Pater J. (1998). Interpreting the Significance of Changes in Health-Related Quality-of-Life Scores. J. Clin. Oncol..

[36] Ryan J.L., Carroll J.K., Ryan E.P., Mustian K.M., Fiscella K., Morrow G.R. (2007). Mechanisms of Cancer-Related Fatigue. Oncologist.

[37] Bower J.E. (2014). Cancer-Related Fatigue–Mechanisms, Risk Factors, and Treatments. Nat. Rev. Clin. Oncol..

[38] Dhruva A., Dodd M., Paul S.M., Cooper B.A., Lee K., West C., Aouizerat B.E., Swift P.S., Wara W., Miaskowski C. (2010). Trajectories of Fatigue in Patients with Breast Cancer before, during, and after Radiation Therapy. Cancer Nurs..

[39] Mustian K.M., Alfano C.M., Heckler C., Kleckner A.S., Kleckner I.R., Leach C.R., Mohr D., Palesh O.G., Peppone L.J., Piper B.F. (2017). Comparison of Pharmaceutical, Psychological, and Exercise Treatments for Cancer-Related Fatigue: A Meta-Analysis. JAMA Oncol..

[40] Cramp F., Byron-Daniel J. (2012). Exercise for the Management of Cancer-Related Fatigue in Adults. Cochrane Database Syst. Rev..

[41] Donovan K.A., Jacobsen P.B., Andrykowski M.A., Winters E.M., Balducci L., Malik U., Kenady D., McGrath P. (2004). Course of Fatigue in Women Receiving Chemotherapy and/or Radiotherapy for Early Stage Breast Cancer. J. Pain Symptom Manag..

[42] Lipsett A., Barrett S., Haruna F., Mustian K., O’Donovan A. (2017). The Impact of Exercise during Adjuvant Radiotherapy for Breast Cancer on Fatigue and Quality of Life: A Systematic Review and Meta-Analysis. Breast.

[43] Ruiz-Casado A., Álvarez-Bustos A., de Pedro C.G., Méndez-Otero M., Romero-Elías M. (2021). Cancer-Related Fatigue in Breast Cancer Survivors: A Review. Clin. Breast Cancer.

[44] Marconi R., Serafini A., Giovanetti A., Bartoleschi C., Pardini M.C., Bossi G., Strigari L. (2019). Cytokine Modulation in Breast Cancer Patients Undergoing Radiotherapy: A Revision of the Most Recent Studies. Int. J. Mol. Sci..

[45] R Core Team (2022). R: A Language and Environment for Statistical Computing.

[46] Wickham H., Miller E., Smith D. (2022). Haven: Import and Export ‘SPSS’, ‘stata’ and ‘SAS’ Files. https://cran.r-project.org/web/packages/haven/index.html.

[47] Wickham H. (2016). ggplot2: Elegant Graphics for Data Analysis.

