# Supplementary material for: Impact of Exclusive Adjuvant Radiotherapy on Fatigue and Quality of Life in Breast Cancer: A Prospective Clinical Study
Source: Cancers (Basel). 2026 Aug 7;18(16):2532. doi: 10.3390/cancers18162532 (PMC13511236; doi:10.3390/cancers18162532)
Supplement: Supplementary file 1 [file cancers-18-02532-s001.zip › File S1.pdf]

Table of Contents

- Descriptive Statistics Tables
- Statistical Report
- Ethics Committee Certificate

			Global	Pre-radiotherapy	Post-radiotherapy
N			140	70	70
Age		x ± SD	55.77 ± 9.38	55.77 ± 9.42	55.77 ± 9.42
Marital Status	Married/Domestic Partnership	% (CI95%)	77.14 % (69.52 -- 83.32 %)	77.14 % (66.05 -- 85.41 %)	77.14 % (66.05 -- 85.41 %)
Marital Status	Widowed	% (CI95%)	5.71 % (2.92 -- 10.87 %)	5.71 % (2.24 -- 13.79 %)	5.71 % (2.24 -- 13.79 %)
Marital Status	Divorced/Separated	% (CI95%)	5.71 % (2.92 -- 10.87 %)	5.71 % (2.24 -- 13.79 %)	5.71 % (2.24 -- 13.79 %)
Marital Status	Single	% (CI95%)	11.43 % (7.16 -- 17.76 %)	11.43 % (5.91 -- 20.96 %)	11.43 % (5.91 -- 20.96 %)
Level Completed Studies	No education	% (CI95%)	5.71 % (2.92 -- 10.87 %)	5.71 % (2.24 -- 13.79 %)	5.71 % (2.24 -- 13.79 %)
Level Completed Studies	Primary	% (CI95%)	30 % (23.03 -- 38.04 %)	30 % (20.54 -- 41.54 %)	30 % (20.54 -- 41.54 %)
Level Completed Studies	Secondary	% (CI95%)	42.86 % (34.96 -- 51.14 %)	42.86 % (31.94 -- 54.52 %)	42.86 % (31.94 -- 54.52 %)
Level Completed Studies	University	% (CI95%)	21.43 % (15.44 -- 28.94 %)	21.43 % (13.44 -- 32.39 %)	21.43 % (13.44 -- 32.39 %)
Employment Status	Active	% (CI95%)	55 % (46.74 -- 63 %)	55.71 % (44.08 -- 66.75 %)	54.29 % (42.7 -- 65.43 %)
Employment Status	Unemployed	% (CI95%)	12.14 % (7.72 -- 18.59 %)	11.43 % (5.91 -- 20.96 %)	12.86 % (6.91 -- 22.66 %)
Employment Status	Home Care	% (CI95%)	32.86 % (25.63 -- 41 %)	32.86 % (23 -- 44.5 %)	32.86 % (23 -- 44.5 %)
Sporting activities	yes	% (CI95%)	61.87 % (53.58 -- 69.52 %)	61.43 % (49.72 -- 71.95 %)	62.32 % (50.52 -- 72.82 %)

			Global	Pre-radiotherapy	Post-radiotherapy
Sporting activities	no	% (CI95%)	38.13 % (30.48 -- 46.42 %)	38.57 % (28.05 -- 50.28 %)	37.68 % (27.18 -- 49.48 %)
Smoker	yes	% (CI95%)	24.29 % (17.93 -- 32.01 %)	24.29 % (15.75 -- 35.5 %)	24.29 % (15.75 -- 35.5 %)
Smoker	no	% (CI95%)	75.71 % (67.99 -- 82.07 %)	75.71 % (64.5 -- 84.25 %)	75.71 % (64.5 -- 84.25 %)
Weight (Kg)		x ± SD	69.18 ± 14.24	69.24 ± 14.27	69.12 ± 14.31
Size (cm)		x ± SD	1.61 ± 0.05	1.61 ± 0.05	1.61 ± 0.05
BMI		x ± SD	26.42 ± 6.56	26.18 ± 7.34	26.68 ± 5.71
Type of cancer	Ductal	% (CI95%)	0.71 % (0.13 -- 3.93 %)	0 % (0 -- 5.2 %)	1.43 % (0.25 -- 7.66 %)
Type of cancer	Infiltrate	% (CI95%)	98.57 % (94.94 -- 99.61 %)	98.57 % (92.34 -- 99.75 %)	98.57 % (92.34 -- 99.75 %)
Type of cancer	In situ	% (CI95%)	0.71 % (0.13 -- 3.93 %)	1.43 % (0.25 -- 7.66 %)	0 % (0 -- 5.2 %)
Type of cancer	Paget	% (CI95%)	0 % (1.73e-16 -- 2.67 %)	0 % (0 -- 5.2 %)	0 % (0 -- 5.2 %)
TNM		x ± SD	0 ± 0	-	0 ± 0
Time since diagnosis		x ± SD	0 ± 0	0 ± 0	0 ± 0
Treatment	Conservative	% (CI95%)	100 % (97.33 -- 100 %)	100 % (94.8 -- 100 %)	100 % (94.8 -- 100 %)
Fatigue causing anxiety		x ± SD	3.52 ± 2.99	1.66 ± 1.33	5.39 ± 3.04
Interference with work/study		x ± SD	3.5 ± 3.07	1.61 ± 1.24	5.39 ± 3.2
Interference with social life		x ± SD	3.14 ± 3.03	1.47 ± 1.13	4.8 ± 3.4
Interference with sexual activity		x ± SD	3.2 ± 3.11	1.39 ± 0.94	5.01 ± 3.46
Interference with leisure		x ± SD	3.28 ± 3.07	1.41 ± 1.01	5.14 ± 3.31
Fatigue intensity/severity		x ± SD	3.52 ± 3.03	1.56 ± 1.24	5.49 ± 3.03
Fatigue pleasantness		x ± SD	3.73 ± 3.21	1.59 ± 1.38	5.87 ± 3.09

		Global	Pre-radiotherapy	Post-radiotherapy
Fatigue pleasant–unpleasant	x ± SD	3.78 ± 3.24	1.64 ± 1.55	5.91 ± 3.07
Fatigue protective–destructive	x ± SD	3.65 ± 3.1	1.7 ± 1.53	5.6 ± 3.05
Fatigue positive–negative	x ± SD	3.72 ± 3.19	1.66 ± 1.55	5.79 ± 3.06
Current fatigue level	x ± SD	3.52 ± 3.11	1.56 ± 1.39	5.49 ± 3.12
Feeling strong–weak	x ± SD	3.72 ± 3.03	1.77 ± 1.53	5.67 ± 2.91
Feeling awake–sleepy	x ± SD	3.26 ± 2.91	1.51 ± 1.15	5.01 ± 3.08
Feeling lively–apathetic	x ± SD	3.54 ± 3.23	1.7 ± 1.79	5.39 ± 3.3
Feeling fresh–tired	x ± SD	3.75 ± 3.23	1.7 ± 1.76	5.8 ± 3.07
Feeling energetic–no energy	x ± SD	3.86 ± 3.32	1.74 ± 1.75	5.97 ± 3.17
Feeling patient–impatient	x ± SD	3.56 ± 3.12	1.66 ± 1.47	5.46 ± 3.19
Feeling relaxed–irritated	x ± SD	3.46 ± 3.02	1.64 ± 1.36	5.27 ± 3.14
Feeling cheerful–depressed	x ± SD	3.46 ± 3.05	1.64 ± 1.59	5.29 ± 3.07
Ability to concentrate	x ± SD	3.32 ± 2.99	1.59 ± 1.29	5.06 ± 3.19
Memory ability	x ± SD	3.2 ± 2.94	1.63 ± 1.55	4.77 ± 3.16
Clarity of thought	x ± SD	3.06 ± 2.87	1.49 ± 1.2	4.63 ± 3.18
Sensory	x ± SD	3.47 ± 2.87	1.6 ± 1.22	5.35 ± 2.82
Behavioral/Severity	x ± SD	3.49 ± 2.94	1.56 ± 1.1	5.42 ± 2.93
Affective Meaning	x ± SD	3.68 ± 3.04	1.67 ± 1.41	5.69 ± 2.9
Cognitive	x ± SD	3.34 ± 2.81	1.61 ± 1.18	5.08 ± 2.9
PIPER total	x ± SD	3.49 ± 2.83	1.61 ± 1.16	5.37 ± 2.76
Vigorous days/week	x ± SD	0.11 ± 0.65	0.11 ± 0.65	0.11 ± 0.65
Vigorous min/day	x ± SD	2.04 ± 13	1.71 ± 10.39	2.36 ± 15.24
Moderate days/week	x ± SD	0.69 ± 1.78	0.74 ± 1.78	0.63 ± 1.79
Moderate min/day	x ± SD	9.14 ± 21.85	11.14 ± 24.45	7.14 ± 18.85
Walking days/week	x ± SD	3.21 ± 2.89	3.4 ± 2.86	3.03 ± 2.92
Walking min/day	x ± SD	34.1 ± 33.08	35.54 ± 33.57	32.66 ± 32.76
Physical activity level numeric	x ± SD	778.15 ± 1044.88	832.91 ± 1012.4	723.4 ± 1080.9

			Global	Pre-radiotherapy	Post-radiotherapy
Physical activity level	Low	% (CI95%)	87.86 % (81.41 -- 92.28 %)	87.14 % (77.34 -- 93.09 %)	88.57 % (79.04 -- 94.09 %)
Physical activity level	Moderate	% (CI95%)	6.43 % (3.42 -- 11.77 %)	5.71 % (2.24 -- 13.79 %)	7.14 % (3.09 -- 15.66 %)
Physical activity level	High	% (CI95%)	5.71 % (2.92 -- 10.87 %)	7.14 % (3.09 -- 15.66 %)	4.29 % (1.47 -- 11.86 %)
Strenuous activity	Not at all	% (CI95%)	39.57 % (31.82 -- 47.87 %)	63.77 % (51.98 -- 74.1 %)	15.71 % (9.01 -- 25.99 %)
Strenuous activity	A little	% (CI95%)	24.46 % (18.06 -- 32.23 %)	23.19 % (14.81 -- 34.4 %)	25.71 % (16.93 -- 37.03 %)
Strenuous activity	Quite a bit	% (CI95%)	18.71 % (13.1 -- 26 %)	8.7 % (4.05 -- 17.7 %)	28.57 % (19.32 -- 40.05 %)
Strenuous activity	Very much	% (CI95%)	17.27 % (11.89 -- 24.41 %)	4.35 % (1.49 -- 12.02 %)	30 % (20.54 -- 41.54 %)
Long walk	Not at all	% (CI95%)	58.57 % (50.29 -- 66.4 %)	81.43 % (70.77 -- 88.81 %)	35.71 % (25.5 -- 47.41 %)
Long walk	A little	% (CI95%)	16.43 % (11.2 -- 23.45 %)	11.43 % (5.91 -- 20.96 %)	21.43 % (13.44 -- 32.39 %)
Long walk	Quite a bit	% (CI95%)	15.71 % (10.61 -- 22.65 %)	7.14 % (3.09 -- 15.66 %)	24.29 % (15.75 -- 35.5 %)
Long walk	Very much	% (CI95%)	9.29 % (5.51 -- 15.24 %)	0 % (0 -- 5.2 %)	18.57 % (11.19 -- 29.23 %)
Short walk	Not at all	% (CI95%)	73.57 % (65.71 -- 80.18 %)	92.86 % (84.34 -- 96.91 %)	54.29 % (42.7 -- 65.43 %)
Short walk	A little	% (CI95%)	16.43 % (11.2 -- 23.45 %)	5.71 % (2.24 -- 13.79 %)	27.14 % (18.12 -- 38.54 %)
Short walk	Quite a bit	% (CI95%)	10 % (6.05 -- 16.09 %)	1.43 % (0.25 -- 7.66 %)	18.57 % (11.19 -- 29.23 %)
Short walk	Very much	% (CI95%)	0 % (1.73e-16 -- 2.67 %)	0 % (0 -- 5.2 %)	0 % (0 -- 5.2 %)

			Global	Pre-radiotherapy	Post-radiotherapy
Bed/chair rest	Not at all	% (CI95%)	65 % (56.79 -- 72.4 %)	91.43 % (82.53 -- 96.01 %)	38.57 % (28.05 -- 50.28 %)
Bed/chair rest	A little	% (CI95%)	15.71 % (10.61 -- 22.65 %)	7.14 % (3.09 -- 15.66 %)	24.29 % (15.75 -- 35.5 %)
Bed/chair rest	Quite a bit	% (CI95%)	15 % (10.03 -- 21.84 %)	1.43 % (0.25 -- 7.66 %)	28.57 % (19.32 -- 40.05 %)
Bed/chair rest	Very much	% (CI95%)	4.29 % (1.98 -- 9.03 %)	0 % (0 -- 5.2 %)	8.57 % (3.99 -- 17.47 %)
Need help (self-care)	Not at all	% (CI95%)	85 % (78.16 -- 89.97 %)	97.14 % (90.17 -- 99.21 %)	72.86 % (61.46 -- 81.88 %)
Need help (self-care)	A little	% (CI95%)	10.71 % (6.6 -- 16.93 %)	0 % (0 -- 5.2 %)	21.43 % (13.44 -- 32.39 %)
Need help (self-care)	Quite a bit	% (CI95%)	2.86 % (1.12 -- 7.12 %)	1.43 % (0.25 -- 7.66 %)	4.29 % (1.47 -- 11.86 %)
Need help (self-care)	Very much	% (CI95%)	1.43 % (0.39 -- 5.06 %)	1.43 % (0.25 -- 7.66 %)	1.43 % (0.25 -- 7.66 %)
Work/daily limits	Not at all	% (CI95%)	62.86 % (54.61 -- 70.42 %)	87.14 % (77.34 -- 93.09 %)	38.57 % (28.05 -- 50.28 %)
Work/daily limits	A little	% (CI95%)	17.14 % (11.8 -- 24.24 %)	7.14 % (3.09 -- 15.66 %)	27.14 % (18.12 -- 38.54 %)
Work/daily limits	Quite a bit	% (CI95%)	14.29 % (9.44 -- 21.04 %)	4.29 % (1.47 -- 11.86 %)	24.29 % (15.75 -- 35.5 %)
Work/daily limits	Very much	% (CI95%)	5.71 % (2.92 -- 10.87 %)	1.43 % (0.25 -- 7.66 %)	10 % (4.93 -- 19.23 %)
Leisure limits	Not at all	% (CI95%)	67.14 % (59 -- 74.37 %)	91.43 % (82.53 -- 96.01 %)	42.86 % (31.94 -- 54.52 %)
Leisure limits	A little	% (CI95%)	17.86 % (12.4 -- 25.03 %)	5.71 % (2.24 -- 13.79 %)	30 % (20.54 -- 41.54 %)
Leisure limits	Quite a bit	% (CI95%)	8.57 % (4.97 -- 14.38 %)	2.86 % (0.79 -- 9.83 %)	14.29 % (7.95 -- 24.34 %)

			Global	Pre-radiotherapy	Post-radiotherapy
Leisure limits	Very much	% (CI95%)	6.43 % (3.42 -- 11.77 %)	0 % (0 -- 5.2 %)	12.86 % (6.91 -- 22.66 %)
Shortness of breath	Not at all	% (CI95%)	72.86 % (64.95 -- 79.54 %)	94.29 % (86.21 -- 97.76 %)	51.43 % (39.95 -- 62.75 %)
Shortness of breath	A little	% (CI95%)	15 % (10.03 -- 21.84 %)	2.86 % (0.79 -- 9.83 %)	27.14 % (18.12 -- 38.54 %)
Shortness of breath	Quite a bit	% (CI95%)	8.57 % (4.97 -- 14.38 %)	1.43 % (0.25 -- 7.66 %)	15.71 % (9.01 -- 25.99 %)
Shortness of breath	Very much	% (CI95%)	3.57 % (1.53 -- 8.09 %)	1.43 % (0.25 -- 7.66 %)	5.71 % (2.24 -- 13.79 %)
Pain	Not at all	% (CI95%)	56.43 % (48.15 -- 64.36 %)	82.86 % (72.38 -- 89.91 %)	30 % (20.54 -- 41.54 %)
Pain	A little	% (CI95%)	21.43 % (15.44 -- 28.94 %)	14.29 % (7.95 -- 24.34 %)	28.57 % (19.32 -- 40.05 %)
Pain	Quite a bit	% (CI95%)	17.86 % (12.4 -- 25.03 %)	2.86 % (0.79 -- 9.83 %)	32.86 % (23 -- 44.5 %)
Pain	Very much	% (CI95%)	4.29 % (1.98 -- 9.03 %)	0 % (0 -- 5.2 %)	8.57 % (3.99 -- 17.47 %)
Need rest	Not at all	% (CI95%)	62.14 % (53.89 -- 69.75 %)	88.57 % (79.04 -- 94.09 %)	35.71 % (25.5 -- 47.41 %)
Need rest	A little	% (CI95%)	17.14 % (11.8 -- 24.24 %)	5.71 % (2.24 -- 13.79 %)	28.57 % (19.32 -- 40.05 %)
Need rest	Quite a bit	% (CI95%)	16.43 % (11.2 -- 23.45 %)	5.71 % (2.24 -- 13.79 %)	27.14 % (18.12 -- 38.54 %)
Need rest	Very much	% (CI95%)	4.29 % (1.98 -- 9.03 %)	0 % (0 -- 5.2 %)	8.57 % (3.99 -- 17.47 %)
Sleep trouble	Not at all	% (CI95%)	50.36 % (42.15 -- 58.55 %)	73.91 % (62.49 -- 82.81 %)	27.14 % (18.12 -- 38.54 %)
Sleep trouble	A little	% (CI95%)	23.02 % (16.81 -- 30.69 %)	15.94 % (9.14 -- 26.33 %)	30 % (20.54 -- 41.54 %)

			Global	Pre-radiotherapy	Post-radiotherapy
Sleep trouble	Quite a bit	% (CI95%)	15.83 % (10.69 -- 22.8 %)	5.8 % (2.28 -- 13.98 %)	25.71 % (16.93 -- 37.03 %)
Sleep trouble	Very much	% (CI95%)	10.79 % (6.65 -- 17.04 %)	4.35 % (1.49 -- 12.02 %)	17.14 % (10.09 -- 27.62 %)
Weakness	Not at all	% (CI95%)	52.14 % (43.92 -- 60.25 %)	80 % (69.18 -- 87.7 %)	24.29 % (15.75 -- 35.5 %)
Weakness	A little	% (CI95%)	27.14 % (20.46 -- 35.05 %)	15.71 % (9.01 -- 25.99 %)	38.57 % (28.05 -- 50.28 %)
Weakness	Quite a bit	% (CI95%)	16.43 % (11.2 -- 23.45 %)	4.29 % (1.47 -- 11.86 %)	28.57 % (19.32 -- 40.05 %)
Weakness	Very much	% (CI95%)	4.29 % (1.98 -- 9.03 %)	0 % (0 -- 5.2 %)	8.57 % (3.99 -- 17.47 %)
Loss of appetite	Not at all	% (CI95%)	76.43 % (68.75 -- 82.69 %)	92.86 % (84.34 -- 96.91 %)	60 % (48.29 -- 70.67 %)
Loss of appetite	A little	% (CI95%)	15 % (10.03 -- 21.84 %)	7.14 % (3.09 -- 15.66 %)	22.86 % (14.59 -- 33.95 %)
Loss of appetite	Quite a bit	% (CI95%)	7.86 % (4.44 -- 13.52 %)	0 % (0 -- 5.2 %)	15.71 % (9.01 -- 25.99 %)
Loss of appetite	Very much	% (CI95%)	0.71 % (0.13 -- 3.93 %)	0 % (0 -- 5.2 %)	1.43 % (0.25 -- 7.66 %)
Nausea	Not at all	% (CI95%)	81.43 % (74.18 -- 87 %)	94.29 % (86.21 -- 97.76 %)	68.57 % (56.97 -- 78.24 %)
Nausea	A little	% (CI95%)	15 % (10.03 -- 21.84 %)	5.71 % (2.24 -- 13.79 %)	24.29 % (15.75 -- 35.5 %)
Nausea	Quite a bit	% (CI95%)	2.86 % (1.12 -- 7.12 %)	0 % (0 -- 5.2 %)	5.71 % (2.24 -- 13.79 %)
Nausea	Very much	% (CI95%)	0.71 % (0.13 -- 3.93 %)	0 % (0 -- 5.2 %)	1.43 % (0.25 -- 7.66 %)
Vomiting	Not at all	% (CI95%)	87.14 % (80.59 -- 91.71 %)	95.71 % (88.14 -- 98.53 %)	78.57 % (67.61 -- 86.56 %)

			Global	Pre-radiotherapy	Post-radiotherapy
Vomiting	A little	% (CI95%)	10.71 % (6.6 -- 16.93 %)	4.29 % (1.47 -- 11.86 %)	17.14 % (10.09 -- 27.62 %)
Vomiting	Quite a bit	% (CI95%)	1.43 % (0.39 -- 5.06 %)	0 % (0 -- 5.2 %)	2.86 % (0.79 -- 9.83 %)
Vomiting	Very much	% (CI95%)	0.71 % (0.13 -- 3.93 %)	0 % (0 -- 5.2 %)	1.43 % (0.25 -- 7.66 %)
Constipation	Not at all	% (CI95%)	75.71 % (67.99 -- 82.07 %)	82.86 % (72.38 -- 89.91 %)	68.57 % (56.97 -- 78.24 %)
Constipation	A little	% (CI95%)	12.86 % (8.29 -- 19.41 %)	8.57 % (3.99 -- 17.47 %)	17.14 % (10.09 -- 27.62 %)
Constipation	Quite a bit	% (CI95%)	10.71 % (6.6 -- 16.93 %)	8.57 % (3.99 -- 17.47 %)	12.86 % (6.91 -- 22.66 %)
Constipation	Very much	% (CI95%)	0.71 % (0.13 -- 3.93 %)	0 % (0 -- 5.2 %)	1.43 % (0.25 -- 7.66 %)
Diarrhea	Not at all	% (CI95%)	82.86 % (75.76 -- 88.2 %)	90 % (80.77 -- 95.07 %)	75.71 % (64.5 -- 84.25 %)
Diarrhea	A little	% (CI95%)	10 % (6.05 -- 16.09 %)	8.57 % (3.99 -- 17.47 %)	11.43 % (5.91 -- 20.96 %)
Diarrhea	Quite a bit	% (CI95%)	6.43 % (3.42 -- 11.77 %)	1.43 % (0.25 -- 7.66 %)	11.43 % (5.91 -- 20.96 %)
Diarrhea	Very much	% (CI95%)	0.71 % (0.13 -- 3.93 %)	0 % (0 -- 5.2 %)	1.43 % (0.25 -- 7.66 %)
Fatigue/Tiredness	Not at all	% (CI95%)	46.04 % (37.98 -- 54.32 %)	76.81 % (65.6 -- 85.19 %)	15.71 % (9.01 -- 25.99 %)
Fatigue/Tiredness	A little	% (CI95%)	25.9 % (19.33 -- 33.76 %)	18.84 % (11.35 -- 29.61 %)	32.86 % (23 -- 44.5 %)
Fatigue/Tiredness	Quite a bit	% (CI95%)	15.83 % (10.69 -- 22.8 %)	2.9 % (0.8 -- 9.97 %)	28.57 % (19.32 -- 40.05 %)
Fatigue/Tiredness	Very much	% (CI95%)	12.23 % (7.78 -- 18.71 %)	1.45 % (0.26 -- 7.76 %)	22.86 % (14.59 -- 33.95 %)

			Global	Pre-radiotherapy	Post-radiotherapy
Pain interference	Not at all	% (CI95%)	52.52 % (44.26 -- 60.64 %)	76.81 % (65.6 -- 85.19 %)	28.57 % (19.32 -- 40.05 %)
Pain interference	A little	% (CI95%)	21.58 % (15.56 -- 29.14 %)	18.84 % (11.35 -- 29.61 %)	24.29 % (15.75 -- 35.5 %)
Pain interference	Quite a bit	% (CI95%)	18.71 % (13.1 -- 26 %)	2.9 % (0.8 -- 9.97 %)	34.29 % (24.25 -- 45.96 %)
Pain interference	Very much	% (CI95%)	7.19 % (3.95 -- 12.74 %)	1.45 % (0.26 -- 7.76 %)	12.86 % (6.91 -- 22.66 %)
Concentration difficulty	Not at all	% (CI95%)	69.78 % (61.7 -- 76.8 %)	88.41 % (78.75 -- 94.01 %)	51.43 % (39.95 -- 62.75 %)
Concentration difficulty	A little	% (CI95%)	15.11 % (10.1 -- 21.99 %)	8.7 % (4.05 -- 17.7 %)	21.43 % (13.44 -- 32.39 %)
Concentration difficulty	Quite a bit	% (CI95%)	10.79 % (6.65 -- 17.04 %)	2.9 % (0.8 -- 9.97 %)	18.57 % (11.19 -- 29.23 %)
Concentration difficulty	Very much	% (CI95%)	4.32 % (1.99 -- 9.1 %)	0 % (3.47e-16 -- 5.27 %)	8.57 % (3.99 -- 17.47 %)
Tension	Not at all	% (CI95%)	47.14 % (39.06 -- 55.38 %)	71.43 % (59.95 -- 80.68 %)	22.86 % (14.59 -- 33.95 %)
Tension	A little	% (CI95%)	28.57 % (21.74 -- 36.55 %)	25.71 % (16.93 -- 37.03 %)	31.43 % (21.76 -- 43.03 %)
Tension	Quite a bit	% (CI95%)	15.71 % (10.61 -- 22.65 %)	2.86 % (0.79 -- 9.83 %)	28.57 % (19.32 -- 40.05 %)
Tension	Very much	% (CI95%)	8.57 % (4.97 -- 14.38 %)	0 % (0 -- 5.2 %)	17.14 % (10.09 -- 27.62 %)
Worry	Not at all	% (CI95%)	47.86 % (39.75 -- 56.08 %)	71.43 % (59.95 -- 80.68 %)	24.29 % (15.75 -- 35.5 %)
Worry	A little	% (CI95%)	25.71 % (19.19 -- 33.53 %)	22.86 % (14.59 -- 33.95 %)	28.57 % (19.32 -- 40.05 %)
Worry	Quite a bit	% (CI95%)	13.57 % (8.86 -- 20.23 %)	2.86 % (0.79 -- 9.83 %)	24.29 % (15.75 -- 35.5 %)

			Global	Pre-radiotherapy	Post-radiotherapy
Worry	Very much	% (CI95%)	12.86 % (8.29 -- 19.41 %)	2.86 % (0.79 -- 9.83 %)	22.86 % (14.59 -- 33.95 %)
Irritability	Not at all	% (CI95%)	54.29 % (46.03 -- 62.31 %)	80 % (69.18 -- 87.7 %)	28.57 % (19.32 -- 40.05 %)
Irritability	A little	% (CI95%)	21.43 % (15.44 -- 28.94 %)	17.14 % (10.09 -- 27.62 %)	25.71 % (16.93 -- 37.03 %)
Irritability	Quite a bit	% (CI95%)	14.29 % (9.44 -- 21.04 %)	2.86 % (0.79 -- 9.83 %)	25.71 % (16.93 -- 37.03 %)
Irritability	Very much	% (CI95%)	10 % (6.05 -- 16.09 %)	0 % (0 -- 5.2 %)	20 % (12.3 -- 30.82 %)
Depression	Not at all	% (CI95%)	52.14 % (43.92 -- 60.25 %)	75.71 % (64.5 -- 84.25 %)	28.57 % (19.32 -- 40.05 %)
Depression	A little	% (CI95%)	18.57 % (13 -- 25.82 %)	15.71 % (9.01 -- 25.99 %)	21.43 % (13.44 -- 32.39 %)
Depression	Quite a bit	% (CI95%)	19.29 % (13.61 -- 26.61 %)	8.57 % (3.99 -- 17.47 %)	30 % (20.54 -- 41.54 %)
Depression	Very much	% (CI95%)	10 % (6.05 -- 16.09 %)	0 % (0 -- 5.2 %)	20 % (12.3 -- 30.82 %)
Memory difficulty	Not at all	% (CI95%)	63.57 % (55.34 -- 71.08 %)	85.71 % (75.66 -- 92.05 %)	41.43 % (30.63 -- 53.12 %)
Memory difficulty	A little	% (CI95%)	21.43 % (15.44 -- 28.94 %)	12.86 % (6.91 -- 22.66 %)	30 % (20.54 -- 41.54 %)
Memory difficulty	Quite a bit	% (CI95%)	6.43 % (3.42 -- 11.77 %)	1.43 % (0.25 -- 7.66 %)	11.43 % (5.91 -- 20.96 %)
Memory difficulty	Very much	% (CI95%)	8.57 % (4.97 -- 14.38 %)	0 % (0 -- 5.2 %)	17.14 % (10.09 -- 27.62 %)
Family interference	Not at all	% (CI95%)	60 % (51.72 -- 67.74 %)	78.57 % (67.61 -- 86.56 %)	41.43 % (30.63 -- 53.12 %)
Family interference	A little	% (CI95%)	23.57 % (17.31 -- 31.25 %)	20 % (12.3 -- 30.82 %)	27.14 % (18.12 -- 38.54 %)

			Global	Pre-radiotherapy	Post-radiotherapy
Family interference	Quite a bit	% (CI95%)	12.14 % (7.72 -- 18.59 %)	1.43 % (0.25 -- 7.66 %)	22.86 % (14.59 -- 33.95 %)
Family interference	Very much	% (CI95%)	4.29 % (1.98 -- 9.03 %)	0 % (0 -- 5.2 %)	8.57 % (3.99 -- 17.47 %)
Social interference	Not at all	% (CI95%)	62.86 % (54.61 -- 70.42 %)	87.14 % (77.34 -- 93.09 %)	38.57 % (28.05 -- 50.28 %)
Social interference	A little	% (CI95%)	22.86 % (16.68 -- 30.48 %)	11.43 % (5.91 -- 20.96 %)	34.29 % (24.25 -- 45.96 %)
Social interference	Quite a bit	% (CI95%)	10 % (6.05 -- 16.09 %)	1.43 % (0.25 -- 7.66 %)	18.57 % (11.19 -- 29.23 %)
Social interference	Very much	% (CI95%)	4.29 % (1.98 -- 9.03 %)	0 % (0 -- 5.2 %)	8.57 % (3.99 -- 17.47 %)
Financial difficulties	Not at all	% (CI95%)	75.71 % (67.99 -- 82.07 %)	85.71 % (75.66 -- 92.05 %)	65.71 % (54.04 -- 75.75 %)
Financial difficulties	A little	% (CI95%)	10 % (6.05 -- 16.09 %)	8.57 % (3.99 -- 17.47 %)	11.43 % (5.91 -- 20.96 %)
Financial difficulties	Quite a bit	% (CI95%)	7.14 % (3.93 -- 12.65 %)	2.86 % (0.79 -- 9.83 %)	11.43 % (5.91 -- 20.96 %)
Financial difficulties	Very much	% (CI95%)	7.14 % (3.93 -- 12.65 %)	2.86 % (0.79 -- 9.83 %)	11.43 % (5.91 -- 20.96 %)
Overall health		x ± SD	5.29 ± 1.64	5.96 ± 1.21	4.61 ± 1.75
Quality of life		x ± SD	5.28 ± 1.65	5.96 ± 1.21	4.6 ± 1.76
Global health status/QoL		x ± SD	71.37 ± 27.42	82.62 ± 20.15	60.12 ± 29.18

STATISTICAL REPORT

Based on the data obtained from women treated with radiotherapy, an observational study was conducted with the primary objective of evaluating the relationship between fatigue and oncological treatment. To achieve this, validated instruments were used to allow a comprehensive assessment of the clinical and functional impact of fatigue, including the EORTC-QLQ-C30 questionnaire, the Piper Fatigue Scale-Revised (PFS-R), and the International Physical Activity Questionnaire (IPAQ).

Using this database, the following analyses were developed:

- Descriptive Analysis: A detailed characterization of the sample was performed by calculating proportions for qualitative variables, and measures of central tendency and dispersion for quantitative variables.

- Association with Radiotherapy: Changes in fatigue levels—both overall and across its behavioral, affective, sensory, and cognitive domains—were evaluated to determine their potential relationship with radiotherapy.

Descriptive Analysis

The analysis is based on a sample of 70 patients.

Tables were created to show the descriptive analysis of the demographic variables and the data collected from the different questionnaires before and after radiotherapy.

The methodology followed is detailed below.

METHODOLOGY

Descriptive Analysis

The data have been described using means and standard deviations, as well as medians and interquartile ranges for continuous quantitative variables. For qualitative variables, proportions and their 95% confidence intervals were determined.

Association with Radiotherapy

The effect of time, physical activity level, and global health status on overall fatigue—as well as its behavioral, affective, sensory, and cognitive domains—was determined using a mixed-effects regression model, applying the method proposed by Zuur & Ieno[1].

Initially, the saturated model was fitted, including all the aforementioned factors and second-degree interactions (Beyond the Optimal). On this model, different error distributions were evaluated (Gaussian with an identity link function; Gamma with a log link function), determining the best fit as the one with the lowest AIC (Akaike's Information Criterion) and the best adequacy of the residuals to the assumptions of normality and homoscedasticity.

Next, using the model that showed the best fit, the random structure of the model $[(1 \mid \mathrm{ID})]$ was optimized using the lme4[2] v.1.1-30 and lmerTest[3] v.3.1-3 packages. Finally, the fixed structure of the model was optimized through backward selection, comparing nested models using the AIC. The interpretation of the model was performed by inspecting the coefficients and the ANOVA calculated with the car[4] v.3.1-3 package. The effects of the fixed factors were inspected by estimating the estimated marginal means and the Tukey test using the emmeans[5] v.1.10.7 package.

In all analyses, [insert tool/method if missing] was used. All analyses were performed using R6 v.4.2.2. Data tables were read using the openxlsx[7] v.4.2.8 package (for xlsx files) and/or haven[8] v. [insert version] (for sav files). Graphs were generated using ggplot 2[9] v.4.0.0, ggpubr[10] v.0.6.0, and other functions integrated into the previously mentioned packages.

Association with Radiotherapy

- **Behavioral Fatigue**
- **Affective Fatigue**
- **Sensory Fatigue**
- **Cognitive Fatigue**
- **Total Fatigue**

Based on the fitted model to explain the behavioral domain of fatigue (BEH), the following structure was employed:

$$\mathrm{BEH} \sim \mathrm{Level} + \mathrm{Time} \times \mathrm{QoF} + (1 \mid \mathrm{ID})$$

Fitted to a Gamma distribution with a logarithmic link function and including the patient identifier as a random effect, the model meets the necessary assumptions (normality of residuals, homoscedasticity, absence of multicollinearity, and influential observations).

References

1.Zuur, A. F. & Ieno, E. N. A protocol for conducting and presenting results of regression-type analyses. *Methods in Ecology and Evolution* **7**, 636–645 (2016).

2.Bates, D., Mächler, M., Bolker, B. & Walker, S. Fitting linear mixed-effects models using lme4. *Journal of Statistical Software* **67**, 1–48 (2015).

3.Kuznetsova, A., Brockhoff, P. B. & Christensen, R. H. B. lmerTest package: Tests in linear mixed effects models. *Journal of Statistical Software* **82**, 1–26 (2017).

4.Fox, J. & Weisberg, S. *An R companion to applied regression*. (Sage, 2018).

5.Lenth, R. V. *Emmeans: Estimated marginal means, aka least-squares means*. (2021).

6. R Core Team. *R: A language and environment for statistical computing*. (R Foundation for Statistical Computing, 2022).

7. Schauberger, P. & Walker, A. *Openxlsx: Read, write and edit xlsx files*. (2025).

8. Wickham, H., Miller, E. & Smith, D. *Haven: Import and export 'SPSS', 'stata' and 'SAS' files*. (2022).

9. Wickham, H. *ggplot2: Elegant graphics for data analysis*. (Springer-Verlag New York, 2016).

10. Kassambara, A. *Ggpubr: 'ggplot2' based publication ready plots*. (2026).

Junta de Andalucía
Consejería de Salud y Familias
SERVICIO ANDALUZ DE SALUD

D. ª MÓNICA SALDAÑA VALDERAS COMO SECRETARIA DEL COMITÉ DE ÉTICA DE LA INVESTIGACIÓN DE CÁDIZ

HACE CONSTAR

Que de acuerdo con lo previsto en la Ley 14/2007, de 3 de julio, de investigación biomédica, este CEI ha revisado en su reunión de fecha 28 de enero de 2021 el estudio de investigación promovido por D. Jesús Baltasar González titulado **Relación entre la fatiga y la radioterapia en mujeres con cáncer de mama. Estudio epidemiológico (nº de registro 138.28).**
Versión de protocolo: 02 de 18 de diciembre de 2020.
Versión de hoja de información al paciente y consentimiento informado: versión 02 de 18 de diciembre de 2020.

Y hace constar que el citado proyecto es viable.

Que presenta suficiente rigor metodológico.

Que la evaluación de costes económicos es correcta.

Que con respecto a su vertiente ética el proyecto cumple los requisitos necesarios de idoneidad del protocolo en relación con los objetivos del estudio.

Y que este Comité acepta que dicho estudio sea realizado en los siguientes centros por los siguientes investigadores principales, correspondiendo a la Dirección de los centros implicados determinar si la capacidad y los medios disponibles son apropiados para realizar el estudio.

Centro	Investigador
Unidad del Linfedema del Campo de Gibraltar. Atención Primaria. A.G.S. del Campo de Gibraltar	D. Jesús Baltasar González Rubiño

Y para que así conste, firmo la presente en Cádiz, a 08 de febrero de 2021

Firmado por SALDAÑA VALDERAS MONICA - 31663449Q el día 08/02/2021 con un certificado emitido por AC FNMT Usuarios

HOSPITAL UNIVERSITARIO PUERTA DEL MAR
Avenida Ana de Viya, 21 – 11009 Cádiz
Teléfono 956 00 21 00 www.hupm.com

ETHICS COMMITTEE CERTIFICATE

MS. MÓNICA SALDAÑA VALDERAS, AS SECRETARY OF THE RESEARCH ETHICS COMMITTEE OF CÁDIZ

HEREBY CERTIFIES:

That, in accordance with the provisions of Law 14/2007 of July 3 on Biomedical Research, this Research Ethics Committee (REC), at its meeting on January 28, 2021, reviewed the research study promoted by Mr. Jesús Baltasar González, titled *"Relationship between fatigue and radiotherapy in women with breast cancer. An epidemiological study"* (Registration No. 138.28).

- **Protocol Version:** 02, dated December 18, 2020.
- **Patient Information Sheet and Informed Consent Version:** Version 02, dated December 18, 2020.

And hereby certifies:

- That the aforementioned project is viable.
- That it presents sufficient methodological rigor.
- That the economic cost assessment is correct.
- That, regarding its ethical dimension, the project meets the necessary requirements for protocol suitability in relation to the study objectives.

And that this Committee approves the performance of said study at the following centers by the following principal investigators, leaving it to the Management of the involved centers to determine if the capacity and available resources are appropriate to carry out the study:

**Research Center**	**Investigator**
**Lymphedema Unit of Campo de Gibraltar.**<br><br>Primary Care. A.G.S. (*Health Care Area*) of Campo de Gibraltar	Mr. Jesús Baltasar González Rubiño

And in witness whereof, I sign this document in Cádiz, on February 08, 2021.

Signed by SALDAÑA VALDERAS MONICA - 31663449Q on 08/02/2021 with a certificate issued by AC FNMT Usuarios (Spanish Royal Mint User Certification Authority).
