# Supplementary material for: Impact of Exclusive Adjuvant Radiotherapy on Fatigue and Quality of Life in Breast Cancer: A Prospective Clinical Study
Source: Cancers (Basel). 2026 Aug 7;18(16):2532. doi: 10.3390/cancers18162532 (PMC13511236; doi:10.3390/cancers18162532)
Supplement: Supplementary file 1 [file cancers-18-02532-s001.zip › Table S1.pdf]

Supplementary Materials

Table S1. Analysis of Variance (ANOVA) summary for generalized linear mixed-effects models evaluating factors associated with cancer-related fatigue.

**Fatigue domain**	**Time (Pre/Post RT)**	**Quality of Life**	**Physical activity level**	**Time × Quality of Life**
**Behavioral**	$\chi^2 = 105.46$ $p < 0.001$	NS	$\chi^2 = 6.32$ $p = 0.042$	$\chi^2 = 28.97$ $p < 0.001$
**Affective**	$\chi^2 = 597{,}391.36$ $p < 0.001$	$\chi^2 = 64.04$ $p < 0.001$	NS	$\chi^2 = 122.09$ $p < 0.001$
**Sensory**	$\chi^2 = 151.46$ $p < 0.001$	$\chi^2 = 14.23$ $p < 0.001$	$\chi^2 = 3.68$ $p = 0.055$	$\chi^2 = 32.01$ $p < 0.001$
**Cognitive**	$\chi^2 = 82.80$ $p < 0.001$	$\chi^2 = 38.26$ $p < 0.001$	NS	NS
**Total fatigue**	$\chi^2 = 139.50$ $p < 0.001$	$\chi^2 = 17.70$ $p < 0.001$	$\chi^2 = 2.84$ $p = 0.092$	$\chi^2 = 27.56$ $p < 0.001$

Abbreviations: RT, radiotherapy; NS, not statistically significant. This table supports the omnibus p-values reported in the main text.
